# ROCK inhibitor fasudil reduces the expression of inflammatory factors in LPS-induced rat pulmonary microvascular endothelial cells via ROS/NF-κB pathway

**DOI:** 10.1186/s40360-022-00565-7

**Published:** 2022-04-15

**Authors:** Huanlong Liu, Zhenhua Pan, Xindi Ma, Junru Cui, Juan Gao, Qingfeng Miao, Zhongning Zhu, Xueyan Chen, Suwen Su

**Affiliations:** 1 Pharmaceutical Department of the Second Hospital of Hebei Medical University, Shijiazhuang, PR China; 2 Department of Pharmacy, Hebei Medical University, Shijiazhuang, PR China; 3 Department of Pharmacology, Hebei Medical University, The Key Laboratory of Neural and Vascular Biology, Ministry of Education, The Key Laboratory of Pharmacology and Toxicology for New Drugs, 361 East Zhongshan Road, Shijiazhuang, 050017 PR China

**Keywords:** Pulmonary microvascular endothelial cells, ROS, NF-κB, Inflammation, Lipopolysaccharide, Fasudil

## Abstract

**Background:**

Inflammation plays a major role in the pulmonary artery hypertension (PAH) and the acute lung injury (ALI) diseases. The common feature of these complications is the dysfunction of pulmonary microvascular endothelial cells (PMVECs). Fasudil, the only Rho kinase (ROCK) inhibitor used in clinic, has been proved to be the most promising new drug for the treatment of PAH, with some anti-inflammatory activity. Therefore, in the present study, the effect of fasudil on lipopolysaccharide (LPS)-induced inflammatory injury in rat PMVECs was investigated.

**Methods:**

LPS was used to make inflammatory injury model of rat PMVECs. Thereafter, the mRNA and protein expression of pro-inflammatory factors was evaluated by reverse transcription-polymerase chain reaction (RT-PCR) and enzyme-linked immunosorbent assay (ELISA) assay respectively. Intracellular reactive oxygen species (ROS) levels were measured by the confocal laser scanning system. The activities of superoxide dismutase (SOD), glutathione peroxidase (GSH-Px) and the content of malondialdehyde (MDA) were determined by using commercial kits according to the manufacturer’s instructions. Western blot assay was used to detect the protein expression of nuclear factor kappa B (NF-κB) p65.

**Results:**

Fasudil effectively prevented inflammatory injury induced by LPS, which is manifested by the decrease of pro-inflammatory cytokines interleukin-6 (IL-6) and monocyte chenotactic protein-1 (MCP-1). Meanwhile, fasudil dramatically reduced the levels of ROS and MDA, and also elevated the activities of SOD and GSH-Px. Furthermore, the nuclear translocation of NF-κB p65 induced by LPS was also suppressed by fasudil. Additionally, the ROS scavengers N-Acetylcysteine (N-Ace) was also found to inhibit the nuclear translocation of NF-κB and the mRNA expression of IL-6 and MCP-1 induced by LPS, which suggested that ROS was essential for the nuclear translocation of NF-κB.

**Conclusions:**

The present study revealed that fasudil reduced the expression of inflammatory factors, alleviated the inflammatory and oxidative damage induced by LPS in rat PMVECs via ROS-NF-κB signaling pathway.

## Introduction

Inflammatory pulmonary artery hypertension (PAH), acute lung injury (ALI) and acute respiratory distress syndrome (ARDS) are clinically the life-threatening medical diseases, usually caused by pathogenic factors such as trauma, pneumonia or sepsis. Among all the above diseases, infection is the major cause [1]. To some extent, inflammation is a protective immune response, which helps to clear away the source of infection. However, uncontrolled excessive inflammation will bring about autoimmune damage, especially in sepsis. Inflammatory factor storm is the main mechanism leading to high mortality in critically ill patients [2]. Cytokines released by infiltrating cells can activate the local pro-inflammatory network, lead to unreversible damage of the lung epithelial and endothelial cells [3]. The common feature of all of these disorders is the dysfunction or injury of pulmonary microvascular endothelial cells (PMVECs) [4]. PMVECs are not only the main target of inflammatory attack, but also the fountainhead of the inflammatory mediators. After being activated, PMVECs can produce a variety of inflammatory mediators, such as monocyte chenotactic protein-1 (MCP-1), interleukin-6 (IL-6), interleukin-8 (IL-8) etc. All these pro-inflammatory mediators can influx into the blood vessels and alveolar spaces directly or through the recruitment of other inflammatory cells, and then aggravate the damage of endothelial cells, resulting in the pathological changes of ALI/ARDS [5]. Therefore, the injury of pulmonary endothelial cells, especially the microvascular endothelial cells, has been considered as one of the characteristics of ALI/ARDS.

Bacterial lipopolysaccharide (LPS), a glycoprotein of the outer membrane of Gram-negative bacteria, can elicit strong inflammatory reaction in vivo and damage the PMVECs directly or indirectly [6]. As a strong promoter of inflammatory response, LPS is the key initiator of endothelial dysfunction in sepsis and various complications including ALI/ARDS [7]. Studies have shown that the toll like receptor 4 (TLR4) expressed in endothelial cells under LPS stimulation is recruited into lipid rafts and interacts with different adaptor molecules, thus activating the downstream signaling pathways to induce the production of pro-inflammatory cytokines [8, 9]. However, the specific signaling pathways leading to inflammatory injury of PMVECs remain unclear, and may be variant in different endothelial systems. Recent evidence has shown that oxidative stress plays a significant role in the pathogenesis of ALI [10, 11]. Moreover, oxidative stress is closely connected to inflammation response and plays an important role during the pathogenesis of ALI/ARDS. As a result, the overproduction of reactive oxygen species (ROS) can cause lung cell injury through a variety of mechanisms, including lipid peroxidation accompanied by the formation of vasoactive molecules and pro-inflammatory molecules, as well as enhancing the expression of pro-inflammatory genes through the changes of transcription factors such as nuclear factor (NF)-κB [12, 13]. Therefore, in order to clarify the complex pathogenesis of ALI/ARDS and then propose some effective prevention or treatment strategies, it is of great significance to study the expression change of cytokines and inflammatory mediators during PMVECs injury induced by LPS.

Rho, a small molecule guanosine triposphate (GTP) binding protein, plays a significant role in various kinds of cell functions by binding to downstream target molecules such as ROCK [14]. Studies have shown that ROCK inhibitors not only significantly reduced vascular permeability, but also inhibited leukocyte migration, systemic inflammation and endothelial injury [15, 16]. Fasudil is the only ROCK inhibitor used in clinic, which has certain anti-inflammatory and immunomodulatory effects [17]. It has been reported that fasudil can inhibit systemic inflammation and guard against ALI/ARDS in septic mice [18]. In addition, fasudil also has been proved to be the most promising new drug for the treatment of PAH in the future [19]. However, these data were not completely conclusive, and the specific mechanism of the beneficial effects of fasudil in inflammatory lung injury is still unclear. Therefore, exploring the cellular and molecular biological mechanisms of fasudil against the inflammatory injury will be invaluable. Our previous research showed that LPS up-regulated the mRNA expression of RhoA and ROCK obviously in rat PMVECs. Considering that ROCK is highly expressed in PMVECs and is closely related to inflammatory reaction [16]. So, in the present study, LPS was used to produce the inflammatory injury in rat PMVECs, and then the effect of fasudil on LPS-induced inflammatory injury in rat PMVECs was investigated.

## Methods

### Reagents

Dulbecco’s modified Eagle medium (DMEM) and fetal bovine serum (FBS) were purchased from Invitrogen (Carlsbad, CA, USA). LPS (*Escherichia coli* 055: B5) were purchased from Sigma-Aldrich (St. Louis, MO). LPS was dissolved with serum-free DMEM medium and made into 1 mg / ml stock solution and was frozen at − 20 °C, then diluted to the required concentration (10 μg/ml) before experiment. The ELISA kits of Rat IL-6 and MCP-1 were purchased from Neobioscience Technology Company (Shenzhen, China). Superoxide Dismutase (SOD), Malondialdehyde (MDA) and Glutathione peroxidase (GSH-Px) Assay Kits were purchased from Nanjing Jiancheng Bioengineering Institute (Nanjing, China). 2′, 7′-dichlorodihydrofluorescein diacetate (DCFH-DA) fluorescent probe was purchased from Molecular Probes (Eugene, OR, USA). N-Acetylcysteine(N-Ace) was purchased from Beyotime Institute of Biotechnology (Shanghai, China). The primers of IL-6, TNF-α, MCP-1 and β-actin were purchased from Sangon Biotech Co., Ltd. (Shanghai, China). Antibodies against NF-kB p65, Histone H_2_A and β-actin were purchased from Cell Signal Technology (Beverly, MA). Fluorescent labeling second antibodies against mouse or rabbit were obtained from ROCKLAND. Fasudil hydrochloride Injection (2 ml: 30 mg) was obtained from Tianjin Hongri Co. (Tianjin, China). The nuclear protein and plasma protein extraction kits were purchased from Beyotime Institute of Biotechnology (Shijiazhuang, China). The SV total RNA isolation system, reverse transcription system and Go Taq Master Mixes were all purchased from Promega (Madison, USA). All other reagents were purchased from Sigma unless otherwise specified.

### Cell culture and treatment

Male Wistar rats, weighing 150-180 g, were used for the primary culture of rat PMVECs, which was approved by the university Institutional Animal Care and Use Committee of Hebei Medical University. According to the explant-culture technique described previously, primary PMVECs were separated from the lung of rats with some improvement [20]. Briefly, the rats were anesthetized with 10% pentobarbital sodium, and the edges of the fresh subpleural lung parenchyma were cut into small pieces (~ 1 mm^3^), and then transferred into culturing bottle. Next, DMEM medium containing FBS (20%), heparin (90 μg/ml), penicillin (100 U/ml) and streptomycin (100 μg/ml) was added to the bottle. After incubation in humidified air containing 5% CO_2_ at 37 °C for 60 h, the explants were removed gently. After reaching to confluence, the cells were passaged every 3 or 4 days. PMVECs were identified in the light of morphology and immunocytochemistry staining with CD34 and coagulation factor VII. PMVECs of passages 2-4 were used in the following experiments.

### MTT assay for cell viability

Firstly, effects of LPS on the viability of rat PMVECs were investigated. Briefly, PMVECs seeded in 96-well plates were made quiescent by incubated in serum-reduced DMEM (1% FBS), then were exposed to LPS at different concentrations for different intervals. The PMVECs were then incubated with MTT (5 mg/ml, 20 ml/well) for 4 h at 37 °C. After the supernatant was removed, the coloured formazan crystals were dissolved in 150 ml of DMSO. Finally, the optical density (OD) value was measured at 490 nm using a microplate reader.

### RT-PCR assay for the mRNA expression of IL-6, TNF-α and MCP-1

Quiescent PMVECs were pretreated with or without fasudil (10, 25, 50 mM) for 2 h, and then LPS (10 μg/ml) was added with different duration. The total RNA was extracted from rat PMVECs according to the manufacturer’s instructions using the SV total RNA isolation system. After eluted in RNase-free water, total RNA was reverse transcribed to cDNA according to the manufacturer’s protocol by using AMV reverse transcriptase with an oligo (dT) primer. Thereafter, the mRNA expressions of IL-6, TNF-α and MCP-1 were evaluated by RT-PCR. The primers for IL-6, TNF-α, MCP-1 and β-actin were designed and synthesized by Sangon Biotech (Shanghai) Co., Ltd. The amplification conditions were as follows: 35 cycles of denaturation at 94 °C for 60 s, annealing at 56 °C for 60 s, and polymerization at 72 °C for 60 s. The expression level of β-actin was regarded as the internal reference. All the PCR products were electrophoresed and scanned with a gel image analysis system (American Thermo Forma), and the mRNA expressions of IL-6, TNF-α and MCP-1 were standardized to that of β-actin. The primer sequences of IL-6, TNF-α, MCP-1 and β-actin are listed in Table 1.

**Table 1:** Primers used for PCR analysis

Gene	Forward primer sequence	Reverse primer sequence	Product size (bp)
IL-6	5′-CTTCCAGCCAGTTGCCTTCT-3′	5′-GAGAGCATTGGAAGTTGGGG-3′	496
TNF-α	5′-GCCAATGGCATGGATCTCAAAG-3′	5′-CAGAGCAATGACTCCAAAGT-3′	357
MCP-1	5′-CACCTGCTGCTACTCATTCACT-3′	5′-GTTCTCTGTCATACTGGTCACTTC-3′	349
β-actin	5′-CCAAGGCCAACCGCGAGAAGATGAC-3′	5′-AGGGTACATGGTGGTGGCGCCAGAC-3′	587

### ELISA assay for the concentration of IL-6 and MCP-1

After PMVECs were treated with LPS (10 μg/ml) for different time, the supernatant of PMVECs in each well of the plate was collected and frozen at − 20 °C for subsequent testing. The concentrations of IL-6 and MCP-1 in supernatant were determined using the enzyme linked-immunosorbent assay (ELISA) kits following the protocols provided by the manufacturer strictly. The levels of IL-6 and MCP-1 were calculated respectively from the standard curves and were expressed as pg/ml protein.

### Measurement of the intracellular ROS levels

The average level of intracellular ROS in rat PMVECs was determined by laser confocal microscopy using a fluorescent probe DCFH-DA. After treated with LPS (10 μg/ml) for different time, PMVECs were incubated with 10 μM DCFH-DA in darkness at 37 °C for 30 min. After diffused into the PMVECs, DCF-DA was hydrolyzed into the nonfluorescent 2′7′-dichlorofluorescein (DCFH). Then, the DCFH was oxidized to high fluorescence DCF by ROS. The fluorescent signal of DCF was detected by the confocal laser scanning system equipped with Nikon E2600 eclipse microscope (Wetzlar, Leica, Germany). Argon laser at the excitation wavelength of 488 nm and the emission wavelength of 530 nm was used in this experiment. The fluorescence intensity of DCF represented the level of reactive oxygen species.

### Measurement of oxidative stress related markers

After appropriate treatment, the PMVECs were collected and resuspended in cold Ripa lysate, ultrasonic treated for 30 s on ice, then centrifuged at 3, 000×g for 15 min. Next, the supernatants were collected and the protein concentration was quantified according to the manufacturer’s instructions of the protein assay kit. The activities of SOD, GSH-Px and the content of MDA were determined by using commercial kits according to the manufacturer’s instructions (Nanjing Jiancheng Bioengineering Institute, Nanjing, China).

### Western blotting analysis

Quiescent PMVECs were pretreated with or without fasudil (10, 25, 50 mM) for 2 h, and then LPS (10 μg/ml) was added for different time. After the PMVECs were collected and lysed, the proteins were extracted following the instructions of nuclear protein and plasma protein extraction kits. The protein concentrations were determined using the protein analysis kit. Thereafter, about 40 μg of whole cell lysate was loaded into each lane. The proteins were first separated on 10% SDS-PAGE gel and then transferred to the nitrocellulose membranes through electroblotting for 2 h at 100 V. After being blocked with 5% skimmed milk for 1 h, the nitrocellulose membranes were incubated with the primary antibodies (1: 200 dilution) overnight at 4 °C, and subsequently the secondary antibodies (anti-rabbit or anti-mouse) bound to IRDye700DX and IRDye800CW (1:5000; Rockland, USA) were used to survey the primary antibodies. Then, the protein bands were detected and quantified by using odyssey biocolor infrared imaging system (LI-COR Biosciences, USA). The immunoblots of β-actin and Histone H_2_A were served as the loading control respectively to normalizd the expressions of NF-κB p65 proteins in the whole cell and nuclear protein lysates.

### Statistical analysis

The data are presented as means ± standard deviation (SD). The statistical analysis included one-way analysis of variance (ANOVA) and Dunnet t′s test (SPSS for Windows v11.0). Differences of *P* < 0.05 were considered as statistically significant. All experiments were repeated at least three times.

## Results

### Effects of LPS on the viability of rat PMVECs

**Fig. 1:**
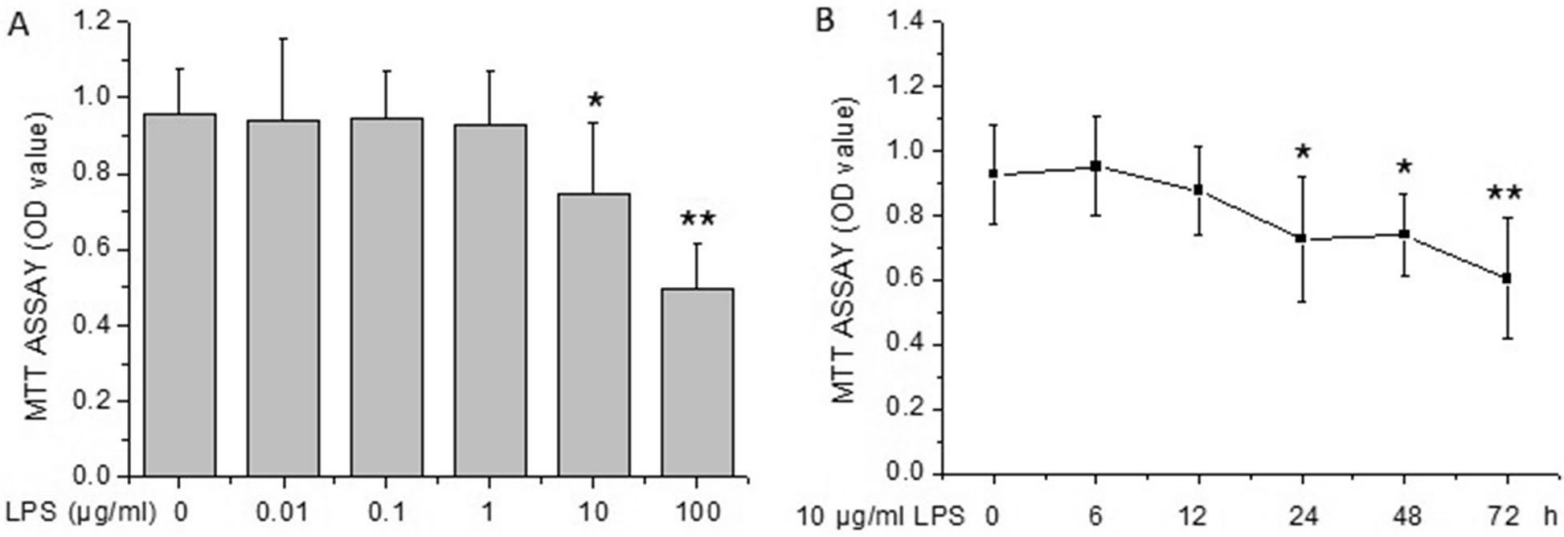
Effect of LPS on the viability of rat PMVECs examined by MTT assay. **A** The cell viability was measured after rat PMVECs were incubated with different concentrations of LPS (0, 0.01, 0.1, 1, 10, and 100 μg/ml) for 24 h. **B** The PMVECs were treated with 10 μg/ml of LPS for indicated durations, then the cell viability was measured by MTT assay. All results were presented as means ± SD of three independent experiments. ^*^P < 0.05, ^**^P < 0.01 vs the value of control group

As shown in Fig. 1A, LPS at 0.01, 0.1 and 1 μg/ml had no significant effect on the viability of rat PMVECs, yet LPS at 10 and 100 μg/ml inhibited the growth significantly. The results demonstrated that 10 μg/ml of LPS reduced the cell viability obviously, but with enough survival cells for the determination of other indexes. So, 10 μg/ml of LPS was selected to the further studies. Next, we observed that the PMVECs began to be destoried significantly after 10 μg/ml of LPS treatment for 24 h, and the effect sustained to 72 h (Fig. 1B).

### Fasudil inhibited LPS-induced inflammatory factors expression in rat PMVECs

**Fig. 2:**
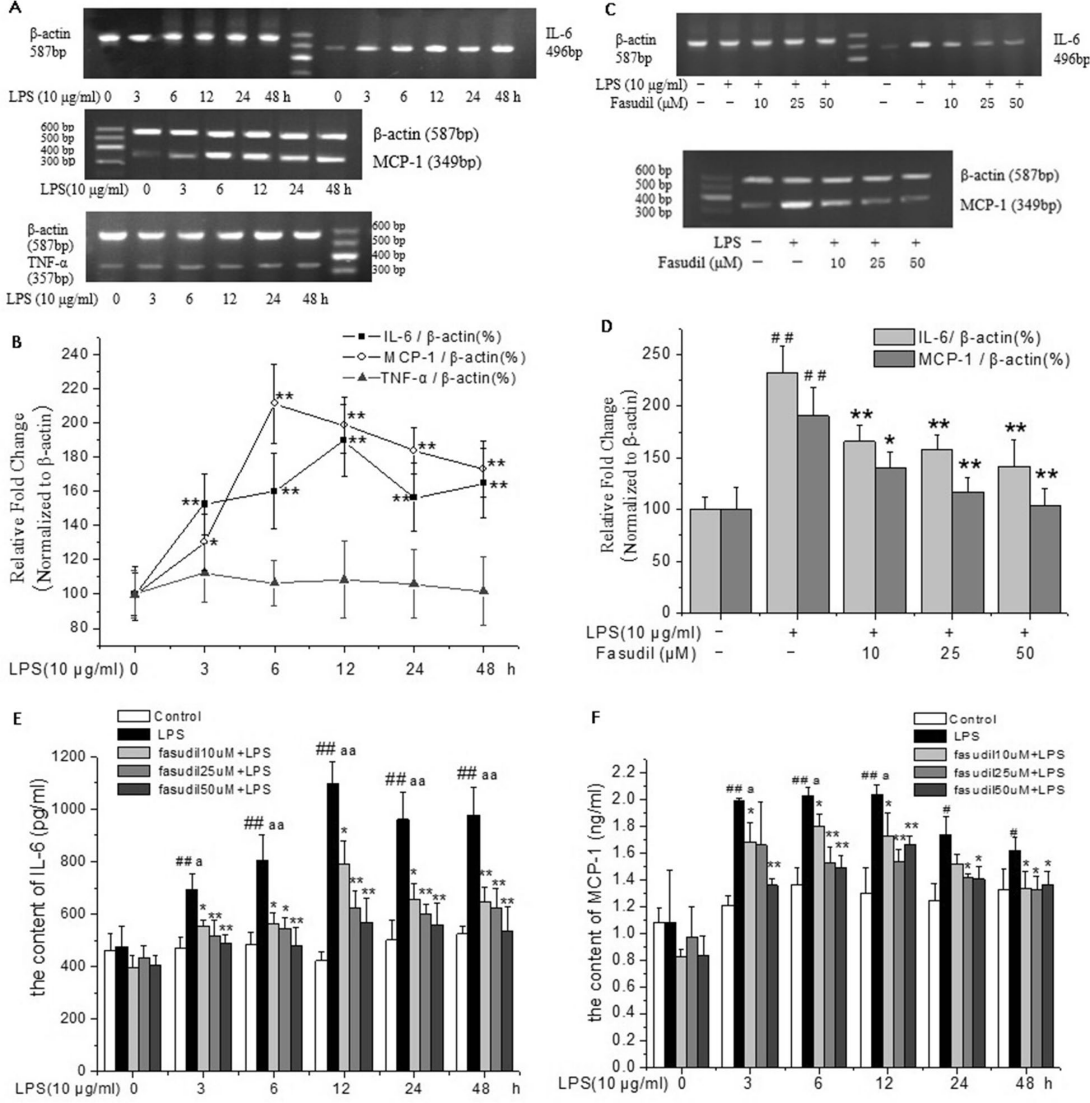
Fasudil inhibited LPS-induced inflammatory factors expression in rat PMVECs. **A** and **B** Effect of LPS (10 μg/ml) on the mRNA expressions of IL-6, TNF-α and MCP-1 in rat PMVECs. Intensity of IL-6, TNF-α and MCP-1 was standardized to that of β-actin respectively. Values were presented as means ± SD of three independent experiments. ^*^P < 0.05, ^**^P < 0.01 versus the value of control group (LPS 0 h). **C** and **D** Fasudil inhibited LPS-induced mRNA expressions of IL-6 and MCP-1 in rat PMVECs. Quiescent PMVECs were incubated with vehicle or fasudil for 2 h, followed by 6 h stimulation with LPS (10 μg/ml) to detect the mRNA expression of MCP-1, and for 12 h stimulation to detect the mRNA expression of IL-6. Intensity of IL-6 and MCP-1 was standardized to that of β-actin. Values were presented as means ± SD of three independent experiments. ^##^*P* < 0.01 vs control group; ^*^P < 0.05, ^**^P < 0.01 vs LPS-alone group. **E** and **F** Fasudil inhibited LPS-induced protein secretion of IL-6 and MCP-1 in the cultural medium of rat PMVECs. Quiescent PMVECs were incubated with vehicle or fasudil for 2 h, followed by stimulation with LPS (10 μg/ml) for indicated durations. The protein concentrations of IL-6 and MCP-1 in culture medium of rat PMVECs were detected respectively by ELISA. Values were presented as means ± SD. ^##^*P* < 0.01 vs the control group (whithout LPS treatment) in each time point; ^*^P < 0.05, ^**^P < 0.01 versus the LPS-alone group in each time point. ^a^*P* < 0.05, ^aa^*P* < 0.01*vs* the group of LPS 0 h

To evaluate the protective effects of Fasudil on LPS-induced inflammatory responses, the mRNA expressions of IL-6, TNF-α and MCP-1 were investigated in rat PMVECs. As shown in Fig. 2A and B, the mRNA expressions of MCP-1 and IL-6 in rat PMVECs were markedly increased upon exposure to LPS. The peak level of expression was arrived at 6 h and 12 h respectively (*P* < 0.01). Whereas, the mRNA expression of TNF-α did not change significantly. Further, the protective effect of fasudil on LPS-induced inflammatory reaction was investigated. Under the same experimental conditions, pre-treatment with fasudil at different concentrations significantly inhibited the increased mRNA expressions of MCP-1 and IL-6 induced by LPS in a concentration-dependent manner (*P* < 0.05 or *P* < 0.01 respectively, Fig. 2C and D). These results suggested that fasudil effectively inhibited LPS-induced inflammation response in rat PMVECs.

To further evaluate the protective effects of fasudil on LPS-induced expression of inflammatory factors, the protein levels of IL-6 and MCP-1 in the supernatant of PMVECs were evaluated by ELISA. The concentration of IL-6 and MCP-1 were increased obviously at each time point of LPS treatment compared with the corresponding control group (*P* < 0.05 or *P* < 0.01, Fig. 2E and F). Under the same experimental conditions, pretreatment with different concentrations of fasudil significantly decreased the increased concentrations of IL-6 and MCP-1 induced by LPS in the supernatant of PMVECs at different time points (*P* < 0.05 or *P* < 0.01 respectively, Fig. 2E and F). These results further confirmed that fasudil could inhibit the inflammatory response induced by LPS in rat PMVECs.

### Fasudil reduced the over-production of ROS induced by LPS in rat PMVECs

**Fig. 3:**
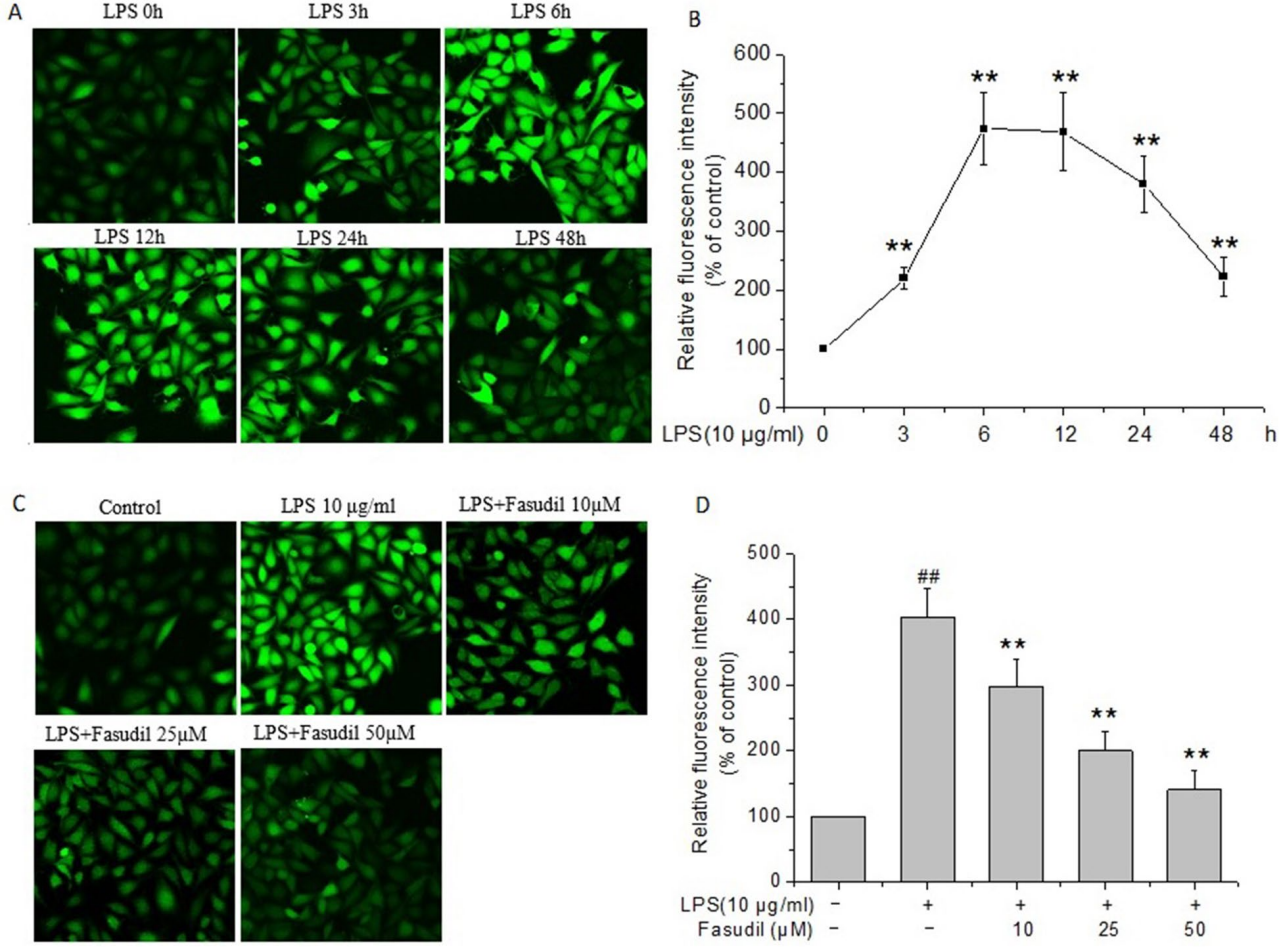
Effect of fasudil on over-production of ROS induced by LPS in rat PMVECs. **A** and **B** LPS induced the production of reactive oxygen species (ROS) in rat PMVECs. Representative fluorescent images of the rat PMVECs and quantitative representation of the relative fluorescence intensity were listed respectively. Values were presented as means ± SD of three independent experiments. ^**^P < 0.01 vs the value at LPS 0 h. **C** and **D** Fasudil inhibited LPS-induced production of ROS in rat PMVECs. Quiescent PMVECs were incubated with vehicle or fasudil for 2 h, followed by stimulation with 10 μg/ml of LPS for 6 h. Then, intracellular ROS level was detected with DCFH-DA staining, visualized by confocal microscopy. Representative fluorescent images and quantitative representation of the relative fluorescence intensity were shown. Values were presented as means ± SD of three independent experiments. ^##^*P* < 0.01 vs control group; ^**^P < 0.01 vs LPS-alone group

To examine whether LPS affects the production of ROS in rat PMVECs, PMVECs were incubated with LPS for different time to detect the ROS levels. As shown in Fig. 3A and B, the ROS levels exhibited obvious increase at 3 h after LPS treatment (*P* < 0.01), with the peak level arriving at 6 h (*P* < 0.01), then grandually decreased during the later observation period to 48 h, but still higher than those of LPS 0 h group (*P* < 0.01). Next, the effect of fasudil on the production of ROS following LPS stimulation was examined. Figure 3C showed the representative fluorescence images of the rat PMVECs at 6 h after LPS stimulation in the presence or absence of fasudil. The statistic results showed that fasudil pretreatment obviously decreased the enhanced fluorescence intensity of ROS induced by LPS (*P* < 0.01, Fig. 3D), which suggested that fasudil significantly inhibited the production of ROS induced by LPS in rat PMVECs.

### Effect of fasudil on oxidative stress-related markers in PMVECs injury induced by LPS

**Fig. 4:**
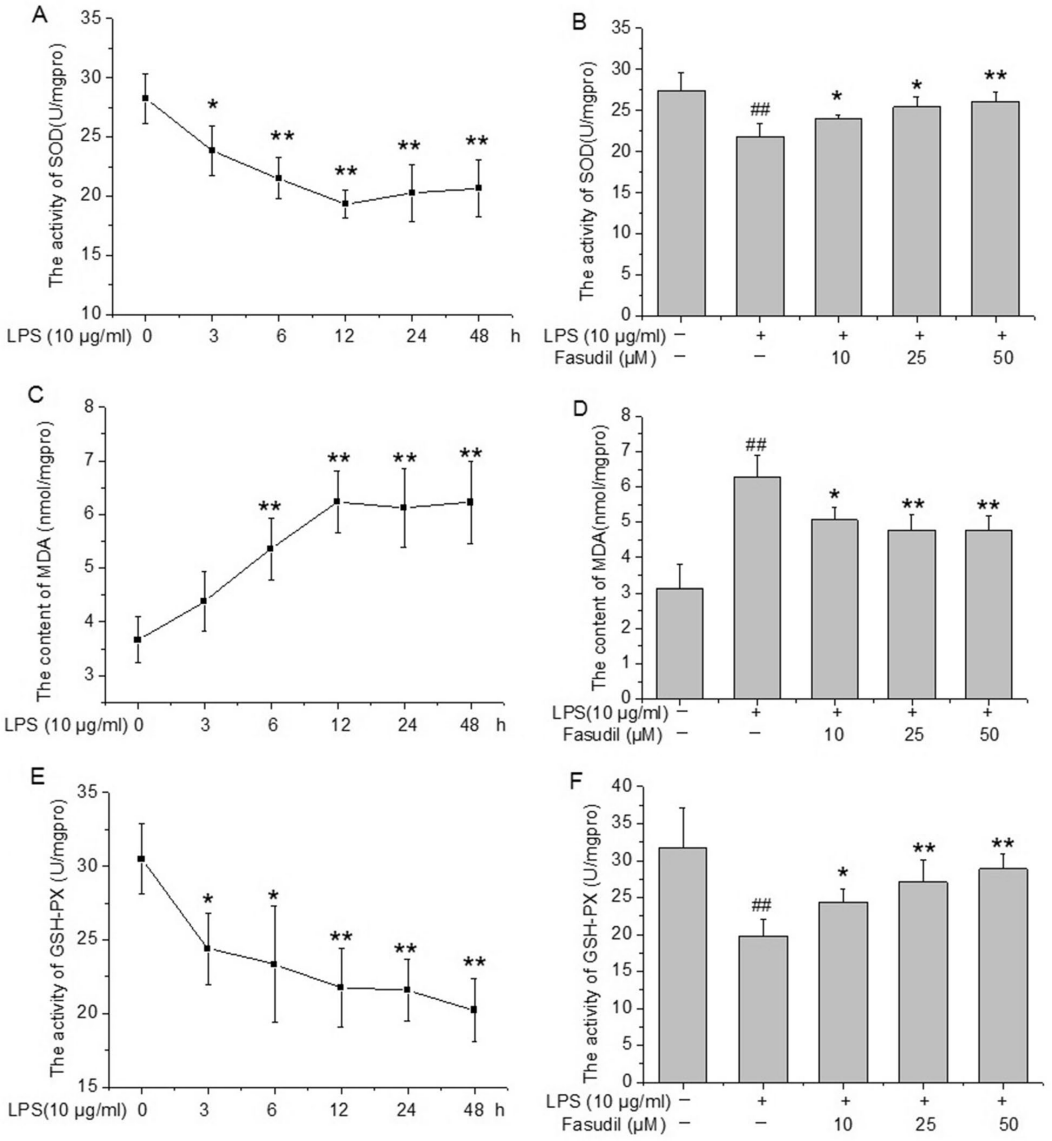
Effect of fasudil on oxidative stress-related markers (SOD, MDA and GSH-Px) in LPS-induced inflammatory injury of rat PMVECs. **A**, **C** and **E** The changes of SOD, MDA and GSH-Px in rat PMVECs after LPS (10 μg/ml) treatment for indicated durations. ^*^P < 0.05, ^**^P < 0.01 vs the value at 0 h. **B**, **D** and **F** Fasudil reversed the changes of SOD, MDA and GSH-Px induced by LPS in rat PMVECs. Quiescent PMVECs were incubated with vehicle or fasudil for 2 h, followed by stimulation with 10 μg/ml of LPS for 12 h. After the cells were lysated, the activities of SOD and GSH-Px and the content of MDA were measured according to the instructions of manufacturer. Values were presented as means ± SD of four independent experiments. ^##^*P* < 0.01 vs control group; ^*^P < 0.05, ^**^P < 0.01 vs LPS-alone group

Oxidative stress is often defined as an imbalance between the production of pro-oxidants and anti-oxidants [21]. Thus, the present study further investigated the effect of fasudil on several oxidative stress biomarkers, including the content of MDA and the activities of two important anti-oxidant enzymes SOD and GSH-Px. Results suggested that significant decreases in the activities of SOD and GSH-Px were observed in rat PMVECs after LPS treatment compared with the control group in a time dependent manner (*P* < 0. 05 or *P* < 0. 01, Fig. 4A and E). Meanwhile, the content of MDA began to increase after LPS treatment from 6 h (*P* < 0.01), then reached the peak level at 12 h and maintained to 48 h (*P* < 0.01 respectively, Fig. 4C). In addition, fasudil (10, 25 and 50 μM) pre-treatment significantly reversed the decreased activities of SOD and GSH-Px and increased content of MDA induced by LPS in rat PMVECs (*P* < 0. 05 or *P* < 0. 01 respectively, Fig. 4B, D and F).

### Effect of fasudil on the nuclear translocation of NF-κB p65 induced by LPS in rat PMVECs

**Fig. 5:**
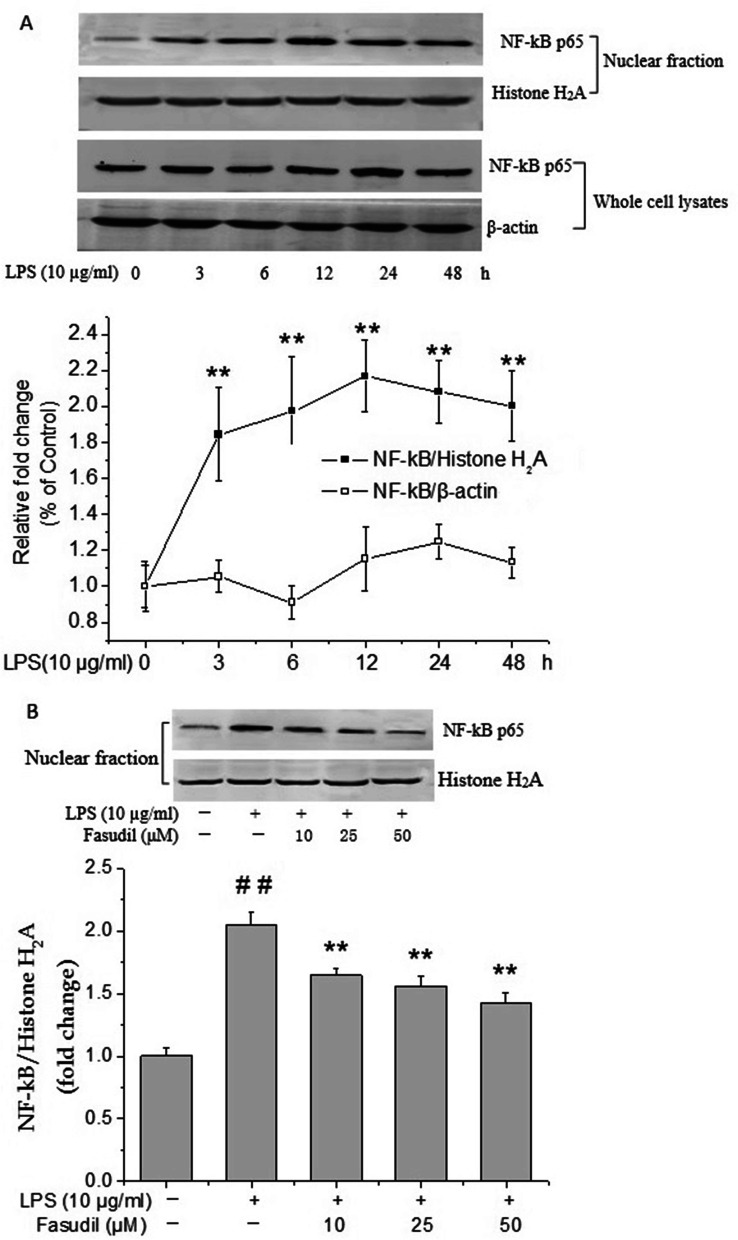
Effect of fasudil on the increased protein expression and nuclear translocation of NF-κB p65 induced by LPS in rat PMVECs. **A** The changes of NF-κB p65 protein expression in the whole cell lysates or nuclear fraction of rat PMVECs after LPS (10 μg/ml) treatment for indicated durations. The β-actin or Histone H_2_A were used for normalization respectively. ^**^P < 0.01 vs the value at 0 h. **B** Fasudil inhibited LPS-induced nuclear translocation of NF-κB p65 in rat PMVECs. Quiescent PMVECs were incubated with vehicle or fasudil for 2 h, followed by stimulation with LPS (10 μg/ml) for 12 h. Western blot images were representative of three independent experiments. Values were presented as means ± SD. ^##^*P* < 0.01 vs control group; ^*^P < 0.05, ^**^P < 0.01 vs LPS-alone group

NF-κB has been reported to play a central role in the antioxidant and anti-inflammatory responses, and also is considered as a critical transcription factor required for the expression of many cytokines [22]. Therefore, the expression of NF-κB was examined by western blot assay. As shown in Fig. 5A, no obvious changes were observed in the protein expression of NF-κB p65 in the whole cell extracts of rat PMVECs after LPS treatment for different time. To further determine the role of NF-κB p65 signaling, the nuclear translocation of NF-κB p65 in LPS treated PMVECs were examined. Results suggested that LPS significantly increased the nuclear translocation of NF-κB p65 as determined by western blot analysis of nuclear-rich cellular fractions (*P* < 0.01, Fig. 5A). NF-κB p65 nuclear translocation occurred transiently with a peak at 12 h. Under the same conditions, fasudil (10, 25 and 50 μM) pretreatment significantly decreased the nuclear translocation of NF-κB p65 induced by LPS in rat PMVECs (*P* < 0.01 respectively, Fig. 5B), which suggested that it might be through inhibiting the nuclear translocation of NF-κB p65 that fasudil reduced the secretion of inflammatory factors.

### Effects of N-Ace on the mRNA expression of inflammatory factors and the nuclear translocation of NF-κB p65 induced by LPS in rat PMVECs

**Fig. 6:**
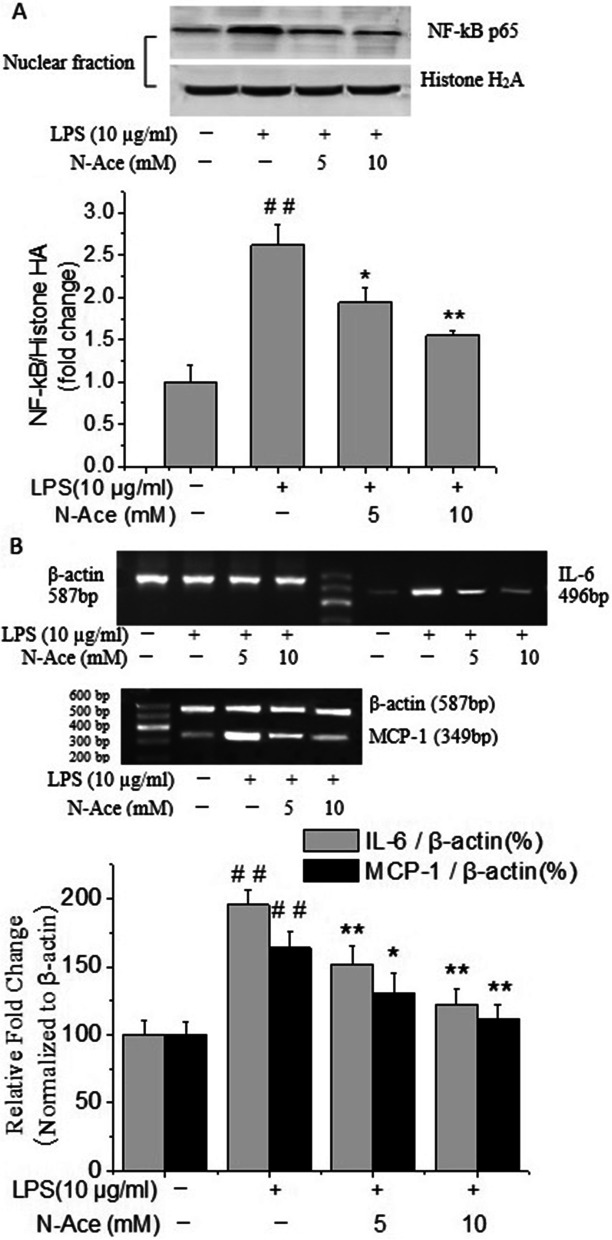
Effects of N-Ace on the increased mRNA expression of inflammatory factors and the nuclear translocation of NF-κB p65 induced by LPS in rat PMVECs. **A** N-Ace inhibited the nuclear translocation of NF-kB p65 induced by LPS in rat PMVECs. Quiescent PMVECs were incubated with vehicle or N-Ace for 2 h, followed by stimulation with LPS (10 μg/ml) for 12 h. Values were presented as means ± SD. ^##^*P* < 0.01 vs control group; ^*^P < 0.05, ^**^P < 0.01 vs LPS-alone group. **B** N-Ace inhibited the increased mRNA expressions of IL-6 and MCP-1 induced by LPS in rat PMVECs. Quiescent PMVECs were incubated with vehicle or N-Ace for 2 h before stimulated with LPS (10 μg/ml) for 6 h or 12 h for the detection of the levels of MCP-1 and IL-6 respectively. Intensity of IL-6 and MCP-1 was standardized to that of β-actin. Values were presented as means ± SD of three independent experiments. ^##^*P* < 0.01 vs control group; ^*^P < 0.05, ^**^P < 0.01 vs LPS-alone group

To further clarify whether increased ROS levels contribute to the inflammatory injury induced by LPS, the effect of reactive oxygen species scavengers on LPS-induced mRNA expression of inflammatory factors and the nuclear translocation of NF-κB p65 in rat PMVECs were assessed. PMVECs were pre-treated with the antioxidant N-Ace and then stimulated with LPS. As shown in Fig. 6A, N-Ace (5 and 10 mM) pre-treatment clearly inhibited the nuclear translocation of NF-κB p65 induced by LPS (*P* < 0.05 or *P* < 0.01 respectively). Furthermore, the increased mRNA expressions of IL-6 and MCP-1 induced by LPS was also markedly decreased by the N-Ace cysteine pretreatment in a concentration dependent manner (*P* < 0.05 or *P* < 0.01 respectively, Fig. 6B). All the results suggested that increased ROS contributes to the inflammatory injury induced by LPS in rat PMVECs.

## Discussion

Although there is a growing understanding of the pathophysiology of ALI / ARDS [23], the mortality of these diseases is still very high, and the therapeutic drugs for these diseases are still deficiency, which reflects that the key events regulating the pathogenesis are still elusive. Recent studies have shown that inflammatory cytokines and chemokines interact to form a complex network, which plays an important role in the pathogenesis of inflammatory lung injury [24, 25]. Considering that inflammation exerts an important role in the occurrence and development of pulmonary inflammatory injury diseases [26], and fasudil has been reported to exert some anti-inflammatory action against various diseases [27, 28]. So, in this study, LPS was used to induce inflammatory response in rat PMVECs, and to explore the cellular and molecular biological mechanism and the protective effect of fasudil. The present study demonstrated that fasudil suppressed the inflammatory response induced by LPS in rat PMVECs, as evidenced by a decrease in the levels of proinflammatory cytokines IL-6 and MCP-1, a reduction in the ROS level and inhibition of the nuclear translocation of NF-κB, which is a pivotal inflammatory mediator after LPS stimulation. So, fasudil protects against the inflammatory and oxidative damage induced by LPS in rat PMVECs by intervening ROS-NF-κB signaling pathway.

As the key parenchymal cells in lung tissue, PMVECs are not only the important target of inflammatory injury, but also the source of inflammatory response [29]. After being activated, PMVECs can produce a lot of cellular chemokines and inflammatory mediators. These inflammatory agents can move directly into the blood vessels and alveolar spaces or move indirectly through the recruitment of other inflammatory cells, thereby aggravate the endothelial cells or surrounding tissues damage [5]. Therefore, the injury of pulmonary vascular endothelial cells, especially pulmonary microvascular endothelial cells, is considered as one of the important characteristics of ALI/ARDS diseases. As the main component of the outer membrane of Gram-negative bacteria, LPS has obvious pro-inflammatory effect and can lead to microvascular endothelial dysfunction to a large extent. Under the action of LPS, PMVECs may suffer from apoptosis, cytoskeleton rearrangement and permeability enhancement [30]. Meanwhile, a large number of inflammatory cytokines will be released to aggravate the PMVECs damage [31]. Therefore, the present study aimed to examine the expression of inflammatory factors in LPS-induced PMVECs injury model. In our study, the mRNA expressions of inflammatory cytokine IL-6 and MCP-1 were increased obviously in rat PMVECs treated with LPS. Whereas, there was no significant change in the mRNA expression of TNF-α, which may be due to that the secretion of inflammatory factors was time-dependent, so that there was no significant change in the time period we observed. The specific reason needs to be further studied. Further, the concentration of IL-6 and MCP-1 in the supernatant of PMVECswere also determined. Results suggested that LPS significantly increased the the concentration of IL-6 and MCP-1 in the supernatant. These data concluded that the inflammation induced by LPS played an important role in the process of PMVECs injury in ALI/ARDS diseases.

Previous studies have shown that the Rho/ROCK signaling pathway plays a significant role in the pathogenesis of sepsis [32]. ROCK inhibitors obviously alleviated the leukocyte migration, systemic inflammation, vascular hyperpermeability and endothelial injury [33, 34]. So, fasudil, a potent and selective ROCK inhibitor, was used in the present study to evaluate its effect on LPS-induced inflammatory damage in rat PMVECs. Our previous study revealed that the phosphorylation level of MYPT1, which represented the degree of ROCK activation, was significantly increased by LPS stimulation in rat PMVECs, fasudil effectively suppressed LPS-induced damage of rat PMVECs through improving cell viability, reducing LDH activity and apoptosis [35]. However, the action and mechanism of fasudil on the inflammatory damage induced by LPS in PMVECs are not fully understood, and the application of fasudil in sepsis remains to be further investigated. So, in the present study, we explored the role of fasudil in PMVECs inflammation induced by LPS, and also elucidated the mechanism of nuclear factor (NF)-κB signalling involved in the process. Our preliminary results suggested that fasudil significantly decreased the increased expressions of inflammatory factors of IL-6 and MCP-1 induced by LPS in rat PMVECs. These results further confirmed that fasudil could inhibit the inflammatory reaction induced by LPS in rat PMVECs.

According to the previous study, fasudil can inhibit systemic inflammation and prevent ALI in sepsis mice [36]. However, the research results are still not very comprehensive and the mechanisms are not yet precisely understood. It is necessary to further study how fasudil achieved these beneficial effects. Reactive oxygen species (ROS), particularly the hydrogen peroxide and superoxide anion, are considered as the vital signal molecules in a lot of biological events [37, 38]. It is well known that various pro-inflammatory substances can activate neutrophils of lungs in patients with ALI/ARDS [39], and the activated neutrophils are the main source of ROS. In addition, oxidative stress injury mediated by ROS in the lung tissue plays an important role in the pathogenesis of ALI/ARDS diseases [40]. There is also recent evidence to show that oxidative stress exerts some significant role in the pathogenesis of ALI [41, 42]. Therefore, excessive production of ROS leads to the modification of many oxidative proteins and results in the acute lung injury. In view of these evidences, this study aimed to determine whether fasudil can ameliorate PMVECs injury induced by LPS via modulating oxidative stress and inflammation. The results of the present study suggested that LPS treatment increased the levels of ROS and MDA (a biochemical marker of peroxidative damage), and decreased the activities of two important anti-oxidant enzymes of SOD and GSH-Px. Fasudil significantly reversed the changes of these oxidative stress indicators induced by LPS. So we concluded that fasudil was effective against inflammation and oxidative stress induced by LPS in rat PMVECs.

ROS can lead to lung injury through a variety of mechanisms, such as DNA damage leading to point mutations and strand breaks. In addition, ROS can also enhance the expression of pro-inflammatory genes by affecting the transcription factors such as nuclear factor NF-κB [43, 44]. To further examine the signaling pathways in LPS-mediated inflammation in rat PMVECs, the role of NF-κB was examined by assessing the protein expression and nuclear translocation of NF-κB p65. As we know, NF-κB is a key transcription factor necessary for the expression of a lot of pro-inflammatory cytokines [45]. Under the stimulation of bacterial components, such as LPS, NF-κB can be activated through a variety of signal transduction mechanisms, resulting in the production of a variety of pro-inflammatory cytokines [46]. Previous studies have shown that NF-κB can be activated by ROCK [47]. In addition, inhibition of ROCK decreased the expression of many inflammatory cytokines and chemokines through regulating the NF-κB signaling pathway [48]. Consistent with the previous findings, our present study also demonstrated that the nuclear translocation of NF-κB p65 was activated in the LPS-induced PMVECs injury, which was antagonized by fasudil, the ROCK inhibitor. Taken together, these data indicated that fasudil protected PMVECs against LPS-induced inflammatory injury, partly due to its potent anti-oxidant and anti-NF-κB p65 nuclear translocation properties.

As noted above, our study revealed that the over-production of ROS level and nuclear translocation of NF-κB p65 were all induced by LPS in rat PMVECs. Whether oxidative stress can promote the expression of inflammatory genes through the changes of transcription factors such as nuclear factor NF-κB in rat PMVECs remains to be further explored. To further clarify these doubts, the ROS scavengers N-Ace was used to study the participation of ROS in the modulation of inflammatory injury induced by LPS in rat PMVECs, and also the contribution of ROS to the nuclear translocation of NF-κB was determined. So, in the present study, rat PMVECs were pretreated with the antioxidant N-Ace and then stimulated with LPS. The results suggested that the N-Ace pretreatment clearly inhibited the nuclear translocation of NF-κB p65 induced by LPS in rat PMVECs. Furthermore, the increased mRNA expressions of IL-6 and MCP-1 induced by LPS were also markedly decreased by the N-Ace pre-treatment. All the data suggested that the ROS play an essential role for the nuclear translocation of NF-κB, and then resulted in the increased expression of inflammatory factors. Based on the experimental results, we can draw a conclusion that fasudil significantly decreased the increased ROS level induced by LPS in rat PMVECs, which might contribute to its anti-inflammatory effect in rat PMVECs.

## Conclusion

In summary, the present study demonstrated that fasudil markedly reduced LPS-induced increase of inflammatory factors including IL-6 and MCP-1 and showed a strong protective effect on inflammatory injury induced by LPS in rat PMVECs. Furthermore, increased ROS level and nuclear translocation of NF-κB induced by LPS were obviously intervened by fasudil pretreatment in rat PMVECs, and increased ROS level was also confirmed to be essential for the nuclear translocation of NF-κB. Therefore, all the results suggested that the protective effect of fasudil on LPS-induced inflammatory injury in rat PMVECs was mediated, at least in part, via the inhibition of ROS-NF-κB signaling pathway.

## Data Availability

The datasets used and/or analyzed during the current study are available from the corresponding author on reasonable request.
